# Targeting the Epigenome in Lung Cancer: Expanding Approaches to Epigenetic Therapy

**DOI:** 10.3389/fonc.2013.00261

**Published:** 2013-10-09

**Authors:** Marko Jakopovic, Anish Thomas, Sanjeeve Balasubramaniam, David Schrump, Giuseppe Giaccone, Susan E. Bates

**Affiliations:** ^1^University of Zagreb, School of Medicine, Department for Respiratory Diseases Jordanovac, University Hospital Center Zagreb, Zagreb, Croatia; ^2^Medical Oncology Branch, Center for Cancer Research, National Cancer Institute, Bethesda, MD, USA; ^3^Surgical Oncology Branch, Center for Cancer Research, National Cancer Institute, Bethesda, MD, USA; ^4^Lombardi Comprehensive Cancer Center, Georgetown University, Washington, DC, USA

**Keywords:** epigenetics, non-small cell lung cancer, small-cell lung cancer, DNA methylation, histone modification, microRNA

## Abstract

Epigenetic aberrations offer dynamic and reversible targets for cancer therapy; increasingly, alteration via overexpression, mutation, or rearrangement is found in genes that control the epigenome. Such alterations suggest a fundamental role in carcinogenesis. Here, we consider three epigenetic mechanisms: DNA methylation, histone tail modification and non-coding, microRNA regulation. Evidence for each of these in lung cancer origin or progression has been gathered, along with evidence that epigenetic alterations might be useful in early detection. DNA hypermethylation of tumor suppressor promoters has been observed, along with global hypomethylation and hypoacetylation, suggesting an important role for tumor suppressor gene silencing. These features have been linked as prognostic markers with poor outcome in lung cancer. Several lines of evidence have also suggested a role for miRNA in carcinogenesis and in outcome. Cigarette smoke downregulates miR-487b, which targets both RAS and MYC; RAS is also a target of miR-let-7, again downregulated in lung cancer. Together the evidence implicates epigenetic aberration in lung cancer and suggests that targeting these aberrations should be carefully explored. To date, DNA methyltransferase and histone deacetylase inhibitors have had minimal clinical activity. Explanations include the possibility that the agents are not sufficiently potent to invoke epigenetic reversion to a more normal state; that insufficient time elapses in most clinical trials to observe true epigenetic reversion; and that doses often used may provoke off-target effects such as DNA damage that prevent epigenetic reversion. Combinations of epigenetic therapies may address those problems. When epigenetic agents are used in combination with chemotherapy or targeted therapy it is hoped that downstream biological effects will provoke synergistic cytotoxicity. This review evaluates the challenges of exploiting the epigenome in the treatment of lung cancer.

## Introduction

Lung cancer is the second most common cancer and leading cause of cancer-related death in both males and females in the United States (1). Traditionally, lung cancer histology allowed it to be divided into two morphologic groups that also demonstrate distinct clinical features: non-small cell lung cancer (NSCLC), which represents around 85% of all cases, and small-cell lung cancer (SCLC). About 80–90% of NSCLCs are directly related to tobacco smoke, while nearly all small cell lung cancers are associated with smoking (2). The vast majority of lung cancers present as advanced and incurable disease at the time of diagnosis (SEER Cancer Statistics Review, 1975–2008); in these patients platinum-based chemotherapy remains a standard treatment for the majority of patients with NSCLC and SCLC, with overall survival (OS) less than 12 months (3, 4). Tailoring chemotherapy according to histologic subset has been shown to improve efficacy in patients with NSCLC (5).

Further progress has been made more recently with improved understanding of the molecular mechanisms involved in oncogenesis (6). These advances enabled the development of drugs that target cancer cell specific gene alterations. These targeted drugs significantly improved response rates and progression-free survival (PFS) in patients with specific genetic alterations (7–9). Despite these advances, the prognosis of patients with both advanced NSCLC and advanced SCLC, remains poor.

Gene expression is regulated by epigenetic mechanisms; epigenetic alterations play important roles in many physiological and pathophysiological conditions, including carcinogenesis, without changes in DNA sequence (10). In addition to genetic alterations in DNA sequence, cancers harbor numerous epigenetic alterations, which regulate gene expression and signaling pathways in the malignant cell. Moreover, these alterations can outnumber genetic alterations and usually occur early in carcinogenesis (11). Numerous data suggest that epigenetics together with genetics intersect to promote carcinogenesis at all stages of cancer development. Increasingly, genomic sequencing of human tumors has identified mutations in genes that encode proteins regulating the epigenome. Epigenetic alterations are in many cases dynamic and reversible, thus representing interesting targets for cancer therapy (12). This review will focus on epigenetic mechanisms of gene regulation in lung cancer and discuss the therapeutic implication of these alterations.

## Types of Epigenetic Changes

Epigenetic mechanisms of gene expression involve various reversible alterations in chromatin structure without changes in nucleotide sequence. Chromatin is the macromolecular complex of DNA and histone proteins that allows packing the entire genome in a single cell. The basic functional unit of chromatin is the nucleosome, an octamer containing two each of the histones H2A, H2B, H3, and H4, around which 146 bp of DNA are wrapped (13). Consecutive nucleosomes are separated by linker DNA, usually 20 and 50 bp in length (14). Classically, nucleosomal DNA is less accessible than linker DNA; the degree of compaction of nucleosomes strongly influences the ability of proteins to target sequences within DNA, modulating transcription, repair, and replication of genes. There are three main types of epigenetic mechanisms: DNA methylation, histone tail modification and non-coding, micro RNA regulation (15).

### DNA methylation

The longest studied epigenetic mechanism of gene expression regulation is the methylation of cytosine residues in CpG sites in the 5′ region of genes. Both DNA hypomethylation and hypermethylation are commonly described in human cancer cells (10). Hypermethylation generally leads to gene silencing and inactivation in tumor suppressor genes, whereas hypomethylation leads to genomic instability and active transcription (15, 16). Global DNA hypermethylation occurs early in the carcinogenesis of lung cancer. Liu et al. showed that hypomethylation of DNA repeats and upregulation of imprinted alleles precede hypermethylation and epigenetic silencing of tumor suppressor genes in epithelial cells exposed to tobacco smoke (17). DNA methylation is mediated via three DNA methyltransferases (DNMT): DNMT1 – 3a and 3b. DNMT1 binds to hemimethylated DNA to maintain methylation patterns after DNA replication (18). On the other hand, DNMTs 3a and 3b bind to unmethylated or hemimethylated DNA to mediate *de novo* DNA methylation (19). Pre-clinical studies have suggested that aberrant expression of DNMTs is involved in carcinogenesis of lung cancer via tumor suppressor gene silencing (20). For example, DNMT1 and DNMT 3b overexpression in lung cancer cells has been correlated with promotor hypermethylation and silencing of the tumor suppressor gene p16 in lung cancer cells (21). Simultaneous overexpression of all three DNMTs and hypermethylation of several tumors suppressor genes including p16, FHIT, and RAR-β was reported by Lin and colleagues (22). Multiple reports have suggested that epigenetic silencing of tumors suppressor genes is involved in the initiation and progression of lung cancer (23–26).

### Modification of histone tails

Lysine-rich tails of core histones (H2A, H2B, H3, and H4) protrude from the nucleosome providing sites for reversible modifications that alter chromatin structure and modulate gene expression (27). These modifications include methylation, acetylation, phosphorylation, sumoylation, and ubiquitination – some of these modifications mark active and some inactive chromatin states (15). The most extensively studied modifications are histone lysine acetylation/deacetylation and methylation/demethylation (27).

Acetylation of histone tails is mediated by a number of histone acetyltransferases (HAT) including GNAT, MYST, and p300 families (27, 28). On the other hand, histone deacetylation is mediated by the histone deacetylase enzymes (HDAC), which are classified in four subfamilies (29). Histone acetylation leads to chromatin relaxation and gene expression, whereas deacetylation leads to gene silencing (30). Non-histone proteins also undergo changes in acetylation state mediated by HATs and HDACs (31).

Numerous histone methyltransferases (KMT) mediate mono, di-, or trimethylation of lysine residues (27). Histone lysine demethylation, on the other hand, is mediated by histone dimethyltransferases (KDMT) (32). Histone methylation may either activate or inhibit gene transcription, depending on the site of action. For example, methylation of lysine 4 on H3 (H3K4) is strongly associated with transcription activation, whereas methylation of lysine 27 on H3 is frequently associated with gene silencing (15). Like histone acetylation, many non-histone proteins such as p53, E2F1, and NFB can be targets of KMT and KDMT (27).

Kim et al showed that increased activity of KMT DOT1L, which mediates methylation of H3K79, supports carcinogenesis of lung cancer cells (33). It is thought that methylation at K79 promotes/inhibits transcriptional elongation, thereby inducing overexpression/underexpression of different cell cycle regulatory genes and different tumor suppressor genes such as HOXA9 and RASSF1A. Overexpression of JARID1B (KDM5B), which demethylates H3K4Me3/Me2, has been observed in both NSCLC and SCLC (34). This overexpression correlated with increased expression of E2F1 and E2F2. Upregulation of LSD1 (KDM1A), which catalyzes demethylation of H3K4Me2/Me1 and possibly H3K9Me2/Me1, was observed in small cell lung cancers relative to normal lung tissues (35). Various alterations in methylation/demethylation and acetylation/deacetylation of core histone in lung cancer cells can be involved in lung carcinogenesis. For example, hyperacetylation of H4K5 and H4K8, hypoacetylation of H4K12/H4K16, and decreased H4K20Me3 levels were observed in lung cancer cells relative to adjacent normal respiratory epithelia (36). It is important to note that these studies report on global changes in expression of specific histone modifications. What is missing is an understanding of which gene promoters are critically affected by these modifications, and which ensuing gene expression changes are involved in carcinogenesis. It is not clear whether changes in histone methylation or acetylation are oncogenic in themselves, or more permissive in allowing the dysregulation of cell growth that is a hallmark of cancer.

### MicroRNAs

MicroRNAs (miRNAs) are small regulatory RNAs, approximately 22 nucleotides long, that control gene expression by binding to the 3′ untranslated region of messenger RNA (mRNA), leading to either mRNA degradation or inhibition of protein translation (37). As negative regulatory factors, miRNAs have emerged as key post-transcriptional regulators of gene expression, involved in many physiological and pathological processes, such as proliferation, differentiation, death, and stress resistance, mediated by altering levels of gene expression (16, 37). There are more than 1,000 mature miRNAs in the human genome according to the miRBase (http://www.mirbase.org/) and it is expected that many more miRNAs will be identified in the future, making their interactions even more complex (15). A single miRNA can target many different mRNAs, and a single mRNA can be targeted by multiple miRNAs, thereby creating a complex network of molecular pathways in cells. Altered expression of miRNAs is commonly found in cancer, and is thus thought to be associated with and potentially contributing to the pathogenesis of most malignancies, including lung cancer, with a miRNA having the potential to serve as either oncogene or tumor suppressor gene, depending on its gene target (38, 39).

The role of the let-7 miRNA family in lung carcinogenesis has been extensively studied. One of the main targets of let-7 miRNA is KRAS (40, 41), leading to its down-regulation. Johnson et al showed that let-7 miRNA expression is lower in lung tumors than in normal lung tissue, while RAS protein is significantly higher in lung tumors, providing a possible mechanistic involvement of let-7 miRNA in carcinogenesis in a subset of lung cancers (40). Hayashita et al. observed that the polycistronic microRNA cluster, miR-17-92, is overexpressed in human lung cancers and enhances cell proliferation and tumor development (42). A recent study demonstrated that loss of miR-365 might also be involved in lung carcinogenesis via decreased suppression of NKX2-1, a transcription factor also known as TTF-1 that is thought to be involved in lung cancer carcinogenesis (43). Enforced NKX2-1 overexpression significantly increased cell proliferation, overcoming the suppressive effect of miR-365 (43).

Xi et al. observed that cigarette smoke repressed miR-487b in cultured respiratory epithelia and lung cancer cells (44). Subsequent experiments revealed significant repression of miR-487b in primary lung cancers – particularly those from smokers, relative to adjacent normal lung tissues. Repression of miR-487b in cultured cells following cigarette smoke exposure and in primary lung cancers coincided with DNA methylation and recruitment of polycomb repressor proteins to the miR-487b regulatory region. Notably, miR-487b directly targets transcripts encoding the non-canonical Wnt ligand, Wnt5a; polycomb repressor proteins BMI1 and SUZ12; and the oncogenes KRAS and MYC. Repression of miR-487b correlated with overexpression of all five transcripts in primary lung cancers. Collectively, these findings demonstrate links between cigarette smoke and the epigenetic repression of a microRNA regulating the expression of five genes involved in oncogenesis.

## Prognostic and Predictive Value of Epigenetic Changes

Early detection of lung cancer could change disease outcome. Until recently, there had not been a screening test that demonstrated a mortality reduction in lung cancer. The National Lung Cancer Screening Trial showed that screening high-risk persons with low-dose CT scanning could significantly reduce lung cancer mortality (45). However, the expense and the potential for harm from even low-dose radiation, raises the question of other approaches. Epigenetic changes develop in smokers and early in lung carcinogenesis, making them potential biomarkers for early detection of lung cancer. Several early but interesting studies have been reported utilizing sputum as a source of tumor cell DNA. Unfortunately, these studies suffer from the problem of “searching under the lamp-post.” As a result there are multiple different epigenetic marks that have been associated with outcome of lung cancer and no comparative analysis that would allow clinical investigators to choose among them for validation studies.

Methylguanine-DNA methyltransferase is a DNA repair enzyme that protects cells from the carcinogenic effects of alkylating agents by removing adducts from the O6 position of guanine; MGMT is frequently inactivated by aberrant promoter methylation in NSCLC (46). Palmisano et al. demonstrated that aberrant methylation of the promoters of the tumor suppressor genes p16 and/or O6-methylguanine-DNA methyltransferase (MGMT) can be detected in DNA from sputum in 100% of patients with squamous cell lung carcinoma up to 3 years before clinical diagnosis (47). Another study confirmed that aberrant promoter hypermethylation of the p16 gene, and to a lesser extent DAP kinase, is detectable in sputum, occurs frequently in smokers and persists after smoking cessation (48).

The melanoma antigen gene (MAGE) is a highly specific tumor marker, and MAGE-A3 expression has been detected in 35% of lung cancer samples and also in pre-cancerous lesions (49, 50). Shin et al. collected sputum from 133 patients with lung diseases (65 lung cancers and 68 benign lung diseases) and showed methylation abnormalities in patients with MAGE-positive sputum (in both malignant and benign diseases). Thus, MAGE expression in the sputum suggests the presence of lung cancer cells or pre-cancerous cells (51).

Interestingly, sputum miRNA profiling using a cluster of five miRNAs (miR-21, miR-143, miR-155, miR-210, and miR-372) detected NSCLC (with 83.3% sensitivity and 100% specificity) in 30 patients. If validated in larger, prospective studies, sputum miRNA profiling could represent a potentially valuable approach for the early detection of NSCLC (52).

## Prognostic Markers

Epigenetic changes have been linked to early recurrence in resected NSCLC. Brock and colleagues reported that DNA methylation in the promoter region of four genes (TP16, CDH13, RASSFIA, and APC) was associated with early recurrence in patients with resected stage I NSCLC (53). Barlési et al. classified 138 lung cancer patients into seven groups based on histology, stage, and global expression levels of H3K4Me2, H2AK5Ac, and H3K9Ac. The groups showed significant differences in disease-free and OS. High intratumoral levels of H3K4Me2 showed significant improvement in OS compared to patients with high intratumoral levels of H3K9Ac (147 vs. 10 months) (54). These changes may help in selecting early-stage NSCLC patients for adjuvant treatment.

DNMT1 accumulation and subsequent hypermethylation of the promoter of tumor suppressor genes may lead to tumorigenesis and provide an important link between tobacco smoking and lung cancer (55). Xing et al. examined DMNT1 and DNMT3b, as well as methylated DNA-binding protein 2 (MBD2) expression in 148 resected NSCLC samples. High DNMT1 expression correlated significantly with increased risk of cancer-related death in all patients, whereas increased DNMT3b expression was associated with poor outcome in patients less than 65 years of age. High-level expression of MBD2 correlated with poor survival in male patients and those with squamous cell carcinomas (56). The previously mentioned study conducted by Kim and colleagues demonstrated that overexpression of DNMT1 is correlated with p16 promoter hypermethylation and diminished patient survival (21). High intranuclear DNMT1 levels correlated significantly with smoking status and poor survival in 124 patients with lung cancer (55). Importantly, DNMT1 overexpression in lung cancer patients who smoked continuously correlated with poor prognosis. The key genes beyond p16 that are hypermethylated and lead to a poor prognosis in patients diagnosed with lung cancer are not known.

Increased HDAC1 mRNA levels appear to be more common in advanced stages of disease in lung cancer patients, thus suggesting a role of HDAC in more aggressive tumors (57). Minamyia et al demonstrated that lung cancer patients with high intratumor levels of HDAC3 had significantly shorter disease-free survivals than patients whose tumors exhibited low HDAC3 expression. They also stated that HDAC3 overexpression was an independent prognostic factor for poor survival in patients with adenocarcinomas, but not in those with squamous cell carcinomas (58). Given that 94 patients were included in this retrospective analysis, confirmatory studies of the role of HDAC3 in NSCLC are needed.

Cellular levels of both H3K4me2 and H3K18ac were reported to predict clinical outcome in lung cancer patients, with lower levels predicting significantly poorer survival (59). Lower global levels of histone modifications are generally predictive of a more aggressive cancer phenotype, revealing a surprising commonality in prognostic epigenetic patterns of adenocarcinomas of different tissue origins, including lung cancer (59).

In a study that analyzed NSCLC and neighboring normal lung tissues, high levels of miR-155 and low miR-let-7a-2 expression were found to correlate with poor survival in lung adenocarcinomas (39). In another study, low let-7 miRNA expression was also significantly associated with shorter survival in 143 resected lung cancer patients (60). Further attempts in addition to sputum analysis, have attempted to find valid biomarkers to detect lung cancer. Interestingly, three separate studies reported that miR-21 overexpression correlated with the aggressiveness of the disease and high levels of miR-21 in serum or plasma were strongly associated with lymph node metastasis, advanced clinical stage, and poor survival in patients with NSCLC (61–63).

## Predictive Markers

A predictive marker is one that may aid in choice among therapeutic options; there are only limited data for predictive epigenetic aberrations in lung cancer patients. Molecular mechanisms of drug resistance are not completely understood and believed to be multifactorial, involving host factors, numerous molecular events, and genetic and epigenetic changes (64). In addition, chemotherapeutics induce epigenetic changes in the promoter area of specific genes, altering expression and possibly underlying resistance in many tumor types (65). One possible reason for the development of chemoresistance in NSCLC might be the epigenetic inactivation of certain tumor suppressor genes as a consequence of chemotherapy treatment (66). Ibanez de Caceres and colleagues reported that a loss of insulin-like growth factor binding protein-3 (IGFBP-3) expression, mediated by promoter hypermethylation, resulted in a reduction of tumor cell sensitivity to cisplatin in NSCLC. Authors suggested that basal methylation status of IGFBP-3 before treatment may be a clinical biomarker and a predictor of chemotherapy outcome, helping to identify patients who are most likely to benefit from platinum-based chemotherapy therapy alone or in combination with epigenetic treatment (66). Expression levels of a miRNA described above as associated with poor outcome, miR-21, were evaluated in tumor tissue and plasma by Gao et al. in 58 patients with resected NSCLC (67). The investigators found increased levels of miR-21 expression in patients with chemotherapy-resistant NSCLC, and concluded that miR-21 may be useful as a biomarker to predict adjuvant platinum-based chemotherapy response and disease-free survival in patients with NSCLC. Thus, it may serve as a novel therapeutic target to modulate platinum-based chemotherapy.

## Targeting the Epigenome

Unlike oncogenic mutations, which are fixed, epigenetic alterations are potentially reversible. The reversibility of epigenetic alterations provides the foundation for targeting them therapeutically. To date, histone deacetylation and DNA methylation have been successfully targeted in the clinic. Several epigenetic modifiers have received FDA approval: DNMT inhibitors, decitabine, and 5-azacitidine are approved for treatment of myelodysplastic syndromes (68, 69) and HDAC inhibitors romidepsin and vorinostat are approved for T-cell lymphoma (70–72).

The adverse effect profiles of these epigenetic drugs are well known from their use for these approved indications. Peripheral cytopenias are among the most common adverse effects of DNMT inhibitors whereas gastrointestinal adverse effects and injection-site reactions are among the most common non-hematological adverse effects. The major adverse effects of HDAC inhibitors are fatigue, nausea, and vomiting. Most of the agents cause thrombocytopenia, lymphopenia, neutropenia, and electrocardiographic changes including ST and T wave flattening and QT prolongation (72, 73). Selective HDAC inhibition may provide greater efficacy and a wider therapeutic window by reducing adverse effects.

## Epigenetic Therapy in NSCLC

**Figure 1:**
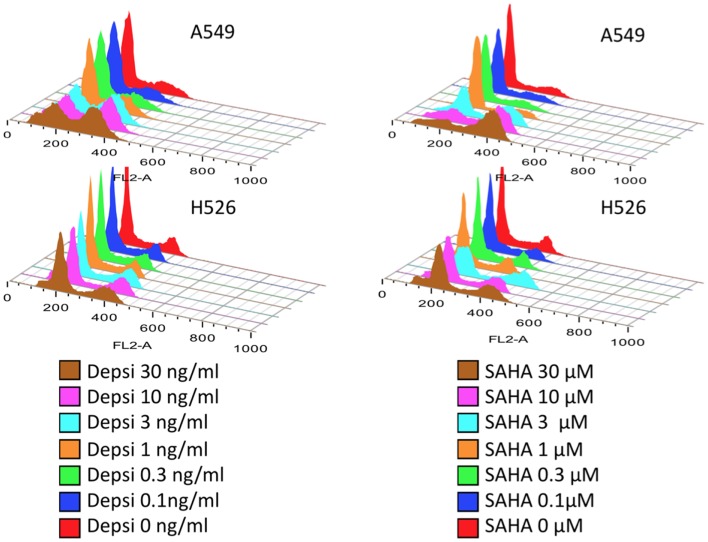
**Lung carcinoma cells treated with HDAC inhibitors**. Dose response studies of cell cycle following 24 h treatment with either romidepsin (DEPSI) or vorinostat (SAHA) are show in the histograms. Concentrations are shown in the legend and represent equipotent concentrations for growth inhibition (Luchenko et al., manuscript in preparation). The NSCLC cell line responds to the HDAC inhibitor with G2 arrest and loss of the G1 peak, while the SCLC line H526 responds with both G1 and G2 arrests and apoptosis.

Table 1 shows the main classes of epigenetic therapies in lung cancer. Table 2 summarizes clinical trials of epigenetic therapies in lung cancer (74–87). Unlike hematological malignancies and CTCL, monotherapy with epigenetic therapies have not proven particularly efficacious in lung cancer, although *in vitro* both romidepsin and vorinostat alone induce apoptosis in NSCLC and SCLC (Figure 1). Available pre-clinical and clinical data suggests that most HDAC inhibitors will be optimally used in combination: with chemotherapies, targeted therapies, radiation, or other epigenetic modifiers, rather than as single agents (88–94).

**Table 1:** **Selected epigenetic drugs which are undergoing clinical evaluation in lung cancer**.

Group	Class	Drug	Mechanism of action
HDAC inhibitors	Aliphatic acids	Valproic acid	Binds to catalytic pocket of lysine deacetylases, complexes with Zn^2+^ via its carboxyl group and inhibits their activity
	Hydroxamic acids	Vorinostat	Pan-HDAC inhibitor
		Belinostat	Pan-HDAC inhibitor
		Panobinostat	Pan-HDAC inhibitor
	Benzamides	Entinostat	Inhibits only the class I enzymes HDAC1 to HDAC3
	Cyclic peptides	Romidepsin	Prodrug whose disulfide bond must be reduced to yield the active form; Inhibitor of class I HDACs
DNA methyl transferase inhibitors	Nucleoside analogs	Decitabine	Phosphorylated form is incorporated in to DNA and inhibits DNA methyltransferase 1
		Azacytidine	Inhibits DNA methyltransferase’s ability to transfer methyl groups to hemimethylated DNA strands
	Small molecules	Hydralazine	Partial competitive inhibitors of DNMT1, decreasing the affinity of DNMT for its substrates

**Table 2:** **Selected clinical trials of epigenetic therapies in lung cancer**.

Drug	Patients	Study design	Enrollment	Drug administration	ORR (%)	Median PFS (m)	Median OS (m)	Author year
**MONOTHERAPY**								
Pivanex	Advanced NSCLC after prior treatment	Single arm phase II	47	2.34 g/m^2^/day 6 h CIVI × 3 days q 21 days	6.4%	1.5	6.2	Reid et al. (78)
CI-994	Advanced NSCLC after prior treatment	Single arm phase II	32	8 mg/m^2^ orally daily	7%	NA	7.5	Wozniak et al. (76)
Vorinostat	Advanced NSCLC after prior treatment	Single arm phase II	16	400 mg orally daily	0	2.3	7.1	Traynor et al. (80)
Panobinostat	SCLC after 1–2 prior treatments	Single arm phase II	21	20 mg/m^2^ iv on day 1 and 8 every 21 days	10%^d^	NR	NR	De Marinis et al. (81)
Decitabine	Advanced NSCLC after prior treatment	Phase I/II	15	200–660 mg/m^2^ IV over 8 h every 21 days	0	NR	6.7	Momparler et al. (75)
Decitabine	Advanced NSCLC after prior treatment	Phase I	35	60–75 mg/m^2^/72 h CIV	0	NR	NR	Schrump et al. (122)
Fazarabine	Advanced NSCLC after prior treatment	Single arm phase II	23	72 h continuous infusion at 2 mg/m^2^/h every 21 days	0	8	NR	Williamson et al. (74)
Romidepsin	Recurrent platinum-sensitive SCLC	Single arm phase II	16	13 mg/m^2^ weekly IV infusions for 3 of 4 weeks	0	1.8	6	Luchenko et al. (93); Otterson et al. (116)
Romidepsin	Recurrent lung cancer	Single arm phase II	19^e^	17.8 mg/m^2^ IV infusions on days 1 and 7 of a 21-day cycle	0	NR	NR	Schrump et al. (79)
**COMBINATION WITH CHEMOTHERAPY**								
Pivanex + docetaxel vs. docetaxel	Advanced NSCLC after prior treatment	Randomized phase II	225	P, 2.5 g/m^2^/day 6 h CIVI × 3 days, D, 75 mg/m 2 day 4	NR^a^	NR	NR	Press release^f^
Gemcitabine + C1-994 or placebo	Advanced NSCLC after prior treatment	Randomized placebo-controlled phase II	180	NA	3.5 vs. 3.8%	NA	6.3 vs. 6.2	Von Pawel et al. (77)
Carboplatin + paclitaxel + Vorinostat or placebo	Advanced NSCLC with no prior treatment	Randomized placebo-controlled phase II	94	V, 400 mg daily or placebo on days 1–14 CP every 3 weeks	34 vs. 12.5%^b^	6 vs. 4.1	13 vs. 9.7	Ramalingam et al. (82)
**COMBINATION WITH TARGETED THERAPIES**								
Erlotinib + Entinostat or placebo^c^	Advanced NSCLC with 1–2 prior chemotherapy	Randomized placebo-controlled phase II	132	E, 150 mg orally daily for 28 days + En, 10 mg orally daily on days 1–15 vs. E + Pl	3 vs. 9.2%	1.9 vs. 1.8	8.9 vs. 6.7	Witta et al. (102)
Bortezomib + Vorinostat	Advanced NSCLC with two prior chemotherapy	Phase II	18	V, 400 mg orally daily on days 1–14 + B, 1.3 mg/m^2^ IV D1, 4, 8, and 11 every 21 days	0	1.43	4.7	Jones et al. (84)
Erlotinib + Vorinostat	Advanced NSCLC with EGFR mutations, progression on erlotinib	Phase I/II	24	E, 150 mg orally daily + V 400 mg orally daily on days 1-7 and 15-21	0	2	10.2	Cardenal et al. (83)
**COMBINATION OF EPIGENETIC THERAPIES**								
Decitabine + Valproic acid	Advanced NSCLC with up to two prior therapy	Phase I	8	De (5–15 mg/m^2^) IV × 10 days + Val (10–20 mg/kg/day) orally on days 5–21 of a 28-day cycle	0	NR	NR	Chu et al. (87)
Azacitidine + Entinostat	Advanced NSCLC after prior treatment	Phase I/II	45	A, 40 mg/m^2^ subcutaneous on days 1–6, 8–10 En, 7 mg orally on days 3, 10 of 28 day cycle	4%	1.9	6.4	Juergens et al. (85)

NSCLC, non-small cell lung cancer; SCLC, small cell lung cancer; P, Pivanex; D, Docetaxel; V, Vorinostat; CP, Carboplatin/Paclitaxel; E, Erlotinib; En, Entinostat; Pl, Placebo; B, Bortezomib; De, Decitabine; Val, Valproic acid; IV, intravenously; CIVI, continuous intravenous infusion; NR, not reported; NA, not available.

^a^ Study halted due to toxicities; development of the agent discontinued.

^b^ Significantly improved ORR in favor of vorinostat arm (*P* = 0.02).

^c^ Primary end-point, 4 month PFS rate which was not significantly different between the groups (Erlotinib + Entinostat, 18% vs. Erlotinib + Placebo, 20%; *P* = 0.7).

^d^ Both responses were not confirmed on follow-up scans.

^e^ Includes 16 NSCLC and 3 NSCLC.

^f^http://www.sec.gov/Archives/edgar/data/910267/000101968704001384/titan_8kex99-1.htm

### Combination with chemotherapy and radiation

Combinations with chemotherapy or radiation therapy and epigenetic agents have been based on the rationale that alterations in gene expression induced by the epigenetic agent may allow increased sensitivity and reverse resistance to treatment. For example, increased topoisomerase II gene expression following HDAC inhibition may increase sensitivity to etoposide (94). Alternately, increased accessibility of DNA due to the “relaxed chromatin” following HDAC inhibition may result in increased platinum binding and increased efficacy. In yet another hypothesis, altered chromatin states resulting from epigenetic alterations have also been identified in small populations of cells that acquire a drug-tolerant phenotype (95). This drug-tolerant subpopulation may be selectively ablated by epigenetic therapies.

Early clinical observations in NSCLC have not supported the pre-clinical findings of synergy between chemotherapy and epigenetic therapy. A randomized placebo-controlled phase II trial of previously untreated NSCLC patients showed improved response rates when vorinostat was added to first-line carboplatin and paclitaxel (34% with vorinostat vs. 12.5% with placebo; *P* = 0.02) (82). However, a phase III randomized trial was prematurely terminated because of no anticipated improvement in response rates, PFS, or OS (96).

These early failures at combining epigenetic therapy with conventional DNA damaging agents may relate to a lack of understanding of the mechanisms underlying the *in vitro* synergy and the conflicting data regarding the optimal schedule for the combination. Whereas some studies suggest that pretreatment with HDAC inhibitors sensitizes cancer cells to chemotherapeutic agents, presumably because sequential treatment may facilitate access to DNA (88–90, 92, 94); other studies are not in agreement with this observation (97, 98). We found that in SCLC cell lines, simultaneous but not sequential treatment with HDAC inhibitors enhanced double-stranded DNA breaks by cisplatin and etoposide. In fact, pretreatment with HDAC inhibitors mitigated the cytotoxicity of DNA damaging agents without increasing their access to DNA (93).

Histone deacetylase enzymes inhibitors have been shown to sensitize NSCLC to the cytotoxic effects of radiation through persistence of DNA double-strand breaks and apoptotic cell death both *in vitro* and *in vivo* (99). The enhanced cytotoxic effects of radiation co-administered with HDAC inhibitors are thought to be due to the effects of HDAC inhibitors decreasing expression or function (via acetylation) of DNA repair proteins (100). Although in theory, HDAC inhibitor induced tumor cell cycle arrest could provide an efficient period for an enhanced response to radiation-induced cell injury, many tumor cells lack the ability to arrest in G1 and by that means to repair their DNA.

### Combination with targeted therapy

Epidermal growth factor receptor (EGFR) tyrosine kinase inhibitors (TKI), erlotinib, and gefitinib are standard treatments for NSCLC and have striking activity against tumors with EGFR mutations (101). However, resistance to the EGFR TKI is observed, and often correlates with markers of epithelial-mesenchymal transition status. Higher levels of E-cadherin, an epithelial marker, indicate sensitivity, whereas higher levels of vimentin and ZEB-1, both mesenchymal markers, indicate resistance. Pre-clinical evidence suggests that HDAC inhibitors can delay as well as reverse EGFR-TKI resistance by inhibiting epigenetic modifications leading to drug tolerance as well as reverting the EMT phenotype (102, 103). A BIM (BCL2L11) deletion polymorphism that results in the generation of alternatively spliced isoforms of BIM that lack the crucial BH3 domain and confers an EGFR TKI resistant phenotype in NSCLC cell lines (104) can be epigenetically restored restored by HDAC inhibition. BIM function is required for apoptosis induction by EGFR-TKIs in EGFR mutant NSCLC (105).

A randomized phase II study that evaluated erlotinib with and without entinostat in previously treated patients with advanced NSCLC and no prior EGFR-TKIs did not find any difference in the primary end-point, 4 month PFS rate between the two groups (Table 2) (102). However, in the subset of patients with high E-cadherin levels, OS was longer in the patients who received erlotinib with entinostat (9.4 vs. 5.4 months; hazard ratio, 0.35; 95% CI, 0.13–0.92; *P* = 0.03). Interim results of a phase I/II study of concurrent administration of vorinostat and erlotinib in patients with advanced NSCLC with EGFR mutations who had prior disease progression on erlotinib showed no objective responses (83). Whether the subset analysis of the erlotinib/entinostat study will be sufficient to move the combination forward with a selection strategy to enroll patients with evidence of EMT in their tumors remains to be determined. Given the *in vitro* evidence we would view the ability to revert EMT as an important question to resolve.

Many kinases, such as EGFR, rely on heat shock protein 90 (Hsp90) chaperone function for conformational maturation and function. HDAC6 deacetylates Hsp90; HDAC6 inhibition results in Hsp90 acetylation, which impairs its chaperone function. This leads to degradation of client proteins. In NSCLC cell lines, HDAC inhibition leads to Hsp90 acetylation, depletion of EGFR, and other key survival signaling proteins, and triggers apoptosis only in lung cancer cells harboring EGFR mutations (103). This mechanism should also provoke synergy with EGFR inhibitors by reducing the level of mutant EGFR that requires inhibition. As noted above, to date few clinical trials have explored this mechanism of synergy, and most unsuccessfully.

Synergistic anti-proliferative effects were seen in pre-clinical models with histone acetylation and proteasome inhibition. HDAC6, a cytoplasmic, microtubule-associated member of the class II family of HDACs plays an essential role in aggresomal protein degradation, a pathway that is upregulated in the setting of proteasome inhibition. Cells that lack HDAC6 do not form aggresomes properly and fail to clear misfolded protein aggregates, which by themselves are toxic (106). HDAC6 inhibition causes the same failure of protein aggregates to traffic to the aggresome. In some cell types, inhibition of proteasome-dependent pathways with bortezomib and the aggresome pathway with HDAC inhibitors leads to a greater accumulation of polyubiquitinated proteins with a resultant increase in cell stress and apoptosis (107). Despite a strong pre-clinical rationale, a phase II study of bortezomib and vorinostat showed no evidence of clinical activity in NSCLC (84).

### Combinations of epigenetic modifiers

DNA methyltransferases inhibitors and HDAC inhibitors in combination have shown synergistic growth inhibition in NSCLC cell lines (108, 109). Clinical studies in patients with hematologic malignancies suggest that sequential administration of DNMT inhibitors and HDAC inhibitors may reverse the silencing of a subset of tumor suppressor genes by a combination of CpG hypermethylation and histone hypoacetylation (110). Initial clinical studies in NSCLC suggest that such combinations may increase clinical efficacy without unacceptable toxicity. A phase I/II trial combined azacitidine and entinostat, in patients with metastatic NSCLC (*n* = 45) (85). One patient had a complete response that lasted 14 months. A second patient had a partial response that lasted 8 months. In the intent-to-treat population, the median PFS was 7.4 weeks (95% CI, 7.0–8.0 weeks) and median OS was 6.4 months (95% CI, 3.8–9.2 months). Median survival among patients who completed at least one cycle of epigenetic therapy was 8.6 months (95% CI, 5.5–12.2 months). One observation in the post-study follow-up of these patients was a major objective response in four out of the 19 patients who received subsequent chemotherapy (21%), which the authors suggested could indicate stable changes in gene expression resulting from epigenetic therapy that altered the cancer cell sensitivity to subsequent cytotoxic therapy.

## Epigenetic Therapy in SCLC

Unlike NSCLC, there is a paucity of data on therapeutic targeting of epigenetic alterations in SCLC. In pre-clinical *in vitro* studies, SCLC has proven sensitive to a number of HDAC inhibitors including panobinostat (111), romidepsin (112), trichostatin (113), and valproic acid (114, 115). Proposed mechanisms of activity of HDAC inhibitors in SCLC include activation of caspases, down-regulation of antiapoptotic factors such as Bcl-2 and Bcl-XL, and upregulation of p21 (111). However, three phase II studies involving monotherapy with two HDAC inhibitors, panobinostat and romidepsin, yielded no objective tumor responses (79, 81, 116).

Several pre-clinical studies of SCLC have shown additive effects of combining epigenetic therapy with chemotherapy (111, 117), and other epigenetic modifiers (118, 119) as well as noting the importance of sequence of administration (93). In an ongoing phase I study, we are evaluating the safety of the combination of belinostat with cisplatin and etoposide in SCLC (NCT00926640). Histone acetylation in peripheral blood mononuclear cells will be determined following belinostat exposure to determine the duration of sustained global acetylation.

## Challenges and Future Directions

Clinical application of epigenetic therapies in lung cancer, and in other tumor types, is still in its early days. Other epigenetic targets such as histone methylation and miRNAs are still in pre-clinical evaluation. There is more to learn about the mechanisms most critical for cancer cell death caused by epigenetic drugs: what are the genes that when demethylated and reexpressed, result in differentiation or cell death? HDAC inhibitors cause global epigenetic changes and some, as we have alluded to above, may act in opposition to promotion of cell death. Further, in lung cancer cell lines and animal models, HDAC inhibition has been shown to enhance cell migration and metastasis through induction of multiple protein kinases and downstream pathways (120). This may diminish therapeutic efficacy, leading to unfavorable outcomes. There is also a lack of understanding of resistance to epigenetic drugs in lung cancer. Although *in vitro* studies have suggested several mechanisms of resistance, they do not typically reflect the insensitivity of solid tumors in the clinic (121). The dual goals of overcoming resistance and the inherent potential of epigenetic drugs to reactivate tumor suppressor genes, point toward the need for rational drug combinations. Although early clinical evidence suggests efficacy of combination therapies in NSCLC, the optimal combinations, sequence, and doses need further study.

The experience with epigenetic drugs in the hematological malignancy setting and the limited experience with solid tumors suggest a delayed response to treatment possibly due to delay between epigenetic effects and ensuing differentiation and cytotoxic effects. For example, the two responses to combination epigenetic therapy in the phase I/II trial that combined azacitidine and entinostat were gradual and maximal response was achieved after only 6–8 months (81). Schrump et al. observed progressive increases in NY-ESO-1 and MAGE-A3 expression in sequential biopsies of an endobronchial tumor from a lung cancer patient treated with Decitabine over a 12-month period (122). NY-ESO-1 and MAGE-A3 protein expression in these biopsies, and serum antibodies recognizing these cancer-testis antigens, were detectable on and after the 6-month timepoint. These as well as other observations from that trial provide molecular evidence that prolonged exposures are necessary to mediate epigenetic alterations in lung cancer cells by DNA demethylating agents. Thus, the conventional strategy of testing drugs in the advanced setting may preclude detection of any clinical benefit from epigenetic drugs in a rapidly growing tumor such as lung cancer. Such delayed benefits may be best detected in the adjuvant setting; a randomized phase II trial comparing 3 year PFS following adjuvant combined epigenetic therapy with azacytidine and entinostat vs. standard care in resected stage I NSCLC (NCT01207726) recently closed. It can also be argued that effectiveness of epigenetic therapy will depend upon its ability to revert the abnormal cancer-related epigenetic changes rather than on direct or indirect cytotoxicity. Hence, the traditional strategy of finding the highest dose that is deemed safe, i.e., the maximum tolerated dose, may not necessarily identify the dose with optimal biological effect. Lastly, identification of lung cancer specific predictive biomarkers is important to identify the appropriate subgroup that may respond to treatment. As noted earlier, multiple biomarkers have been identified and need further evaluation.

Emerging data from large-scale sequencing studies underscore the major role of epigenetics in human cancer and point to a much closer collaboration between genetic and epigenetic events in carcinogenesis (123). Coupled with results of ongoing efforts to map human epigenomes in great detail, this understanding will significantly expand the implications of epigenome in lung cancer detection, prevention, and treatment.

## Conflict of Interest Statement

The authors declare that the research was conducted in the absence of any commercial or financial relationships that could be construed as a potential conflict of interest.
